# Allergy to ustekinumab: Validating skin tests for diagnostic and therapeutic decision-making

**DOI:** 10.5415/apallergy.0000000000000150

**Published:** 2024-07-10

**Authors:** Pedro B. Alves, Francisca Cunha, Sofia Mendes, Carmelita Ribeiro, Ana Todo-Bom

**Affiliations:** 1 Allergy and Clinical Immunology Unit, Coimbra Hospital and University Centre, Coimbra, Portugal; 2 Gastroenterology Department, Coimbra Hospital and University Centre, Coimbra, Portugal

**Keywords:** Desensitization, drug allergy, monoclonals, skin testing

## Abstract

Monoclonal antibodies have gained prominence in treating cancer and inflammatory diseases, but their increased use is linked to rising hypersensitivity reactions. Our case report focuses on a 32-year-old female with Crohn’s disease treated initially with adalimumab and later with ustekinumab. Despite ustekinumab’s generally safe profile, the patient developed increasingly severe mucocutaneous reactions. Intradermal skin testing with a 1:10 (0.5 mg/ml) concentration, validated with negative controls, revealed a type I hypersensitivity reaction to ustekinumab. The patient’s continuous need for the drug led to a desensitization protocol, with a generalized mucocutaneous reaction during the second cycle. This case report underscores the growing concern of monoclonal hypersensitivity and the need for accurate diagnosis and therapeutic adjustments. Skin testing, especially for type I and type IV phenotypes, is valuable but lacks standardized concentrations and accessibility. This report validates nonirritating concentrations for ustekinumab in skin testing for IgE-mediated reactions, a novel contribution to existing literature. Skin testing aided risk stratification and led to the development of a desensitization protocol. A broader application of these skin test concentrations, combined with in vitro testing, could enhance diagnostic accuracy and risk prediction for ustekinumab reactions, presenting skin testing as a promising diagnostic and stratification tool for future use.

## 1. Introduction

The efficacy of monoclonal antibodies (mABs) in the treatment of cancer and inflammatory diseases has led to a significant rise in their use in recent years. This widespread exposure has been associated with an increase in hypersensitivity reactions to mABs [1]. The physiopathology behind these reactions is being extensively studied and has greatly changed recently, differing from the classic Gell and Coombs classification and branching out to 7 different types of mechanisms—from type I immediate reactions to type VII chemical responses [2]. Ustekinumab (*Stelara*) is a fully human IgG1κ mAB against interleukin-12 and -23 that was approved in 2016 by the European Medicines Agency and the US Food and Drugs Administration as a second-line agent for the treatment of moderate to severely active Crohn’s disease [3]. Despite its safe profile, rare hypersensitivity reactions have been reported [4, 5]. We present one such case and our diagnostic work-up approach that highlights the utility of validating skin tests for these rare cases.

## 2. Case report

**Figure 1.**
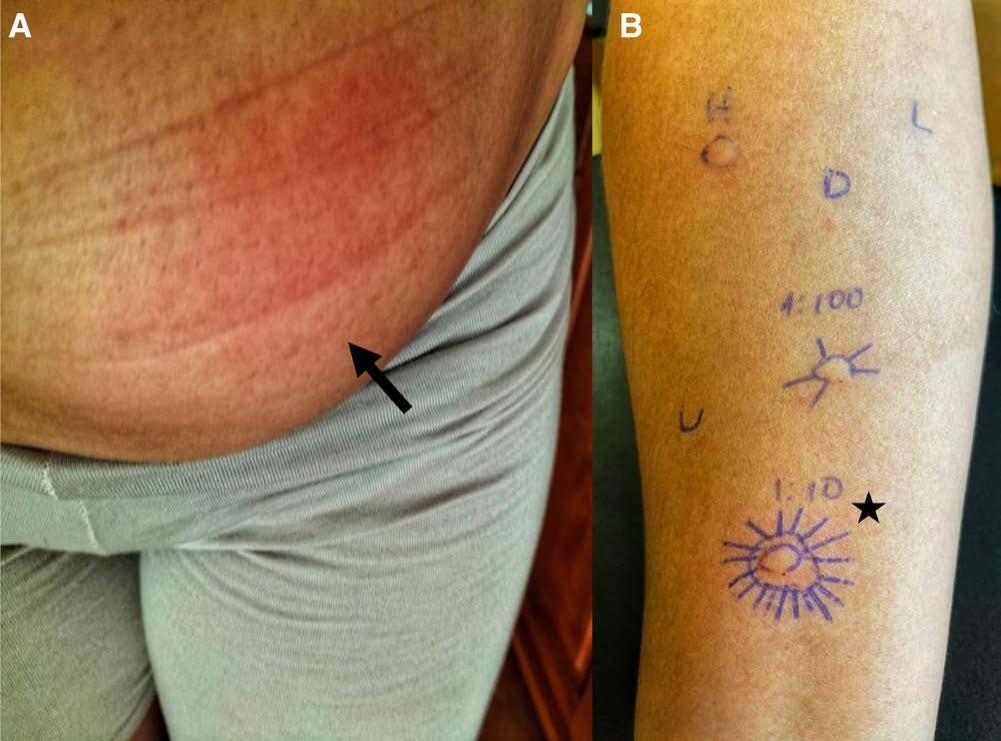
Patient with IgE-mediated reaction to ustekinumab. **(A**) Local reaction, with significant abdominal edema (arrow); **(B**) skin testing to ustekinumab, with positive 0.5 mg/ml intradermal (star). Reaction wheal diameter—9 mm; initial wheal—5 mm. D, normal saline; H, histamine; L, latex extract; U, 1:1 prick test.

Our patient is a 32-year-old female with a history of allergic rhinitis and severe Crohn’s disease (ileo-colic pattern—A2L3B1), associated with spondylarthritis, diagnosed in 2017. Due to the inefficacy of treatment with budesonide, the patient had been previously treated with subcutaneous adalimumab in 2020. However, she discontinued treatment after 10 months due to local reactions on the injection site (edema and induration) and elevated antiadalimumab antibodies (91.1 ng/ml; reference levels <10 ng/ml) with nontherapeutic drug levels (0.3 μg/ml; therapeutic interval 5–12 μg/ml) [6]. Intravenous treatment with ustekinumab 390 mg (5 mg/ml concentration) was then initiated in 2021, followed by subcutaneous 8-week maintenance dosing intervals (90 mg–90 mg/ml concentration). The maintenance protocol was well tolerated until March 2023, when the patient reported local pruritus, erythema, and edema 10 minutes after administration, followed by increased swelling in the injection site (Fig. 1A). The 3 subsequent cycles were associated with symptom progression—in May and July, local pain and an increase in the edema’s extension; in September, aggravation of previous symptoms and generalized pruritus immediately after administration. The patient was treated with clemastine 2 mg and/or hydrocortisone 100 mg at the gastroenterology’s day hospital during this treatment cycle, with immediate relief and symptom resolution after 3 days. No systemic symptoms were reported at the time.

She was then referred to our allergy clinic for a diagnostic work-up, 6 weeks after the last reaction. We conducted a 1:1 skin prick test and intradermal testing with ustekinumab (5 mg/ml) at increasing concentrations of 1:100, 1:10, and 1:1. Prick testing with latex and positive (histamine 10 mg/ml) and negative (normal saline) controls were also performed. A positive reaction was observed with intradermal testing at 1:10 concentration (0.5 mg/ml) of ustekinumab, with erythema, infiltration, and intense pruritus (Fig. 1B). The excipient polysorbate-80, which is present in the drug’s composition, was also tested with negative results, and the patient also tolerated drugs with this component (eg, vaccination with flu virus vaccine Influvac Tetra). In compliance with the 2002 allergy recommendations for skin test diagnostic procedures, we then excluded irritant reactions by testing the intradermal 1:10 concentration in 10 nonexposed controls [7]. No reactions were observed in this group. These results confirmed the diagnosis of type I hypersensitivity to ustekinumab.

Due to a lack of viable alternatives and considering its efficacy in controlling concomitant arthritis complications, a collective decision was reached with both the patient and the gastroenterology department to maintain the ongoing treatment with ustekinumab. A medically-supervised drug administration and premedication regimen with montelukast 10 mg, cetirizine 10 mg, and methylprednisolone 60 mg was subsequently performed but did not prevent another allergic reaction, with quicker onset, papular exanthema, and a more intense generalized pruritus. To prevent systemic reactions, an 11-step and 3-bag desensitization protocol for ustekinumab 390 mg was then conducted, using the intravenous formulation of the drug and based on previous publications on mABs [3, 5]. The first cycle was carried out successfully, with no adverse systemic events. However, after 8 weeks, using the same protocol and during the final step of the second cycle (5 mg/ml concentration, at an infusion rate of 40 ml/h), the patient presented with generalized pruritus and urticaria, and bilateral palpebral angioedema. No other systemic symptoms were reported. The reaction was successfully treated with intravenous clemastine 2 mg and hydrocortisone 100 mg, and infusion was resumed after 30 minutes at 20 ml/h with no further issues. Acute tryptase levels were measured during this reaction, with negative results (6.3 ng/ml).

## 3. Discussion

Hypersensitivity to mABs is an increasingly prevalent issue in allergy clinics due to their widespread use and multiple administration schemes that can lead to sensitization. Their efficacy and increasing dependency as first- and second-line treatments for neoplastic and inflammatory diseases reinforce the need to properly diagnose potential hypersensitivity reactions and accordingly plan adjustments to the therapeutic scheme (eg, desensitization and/or premedication protocols) [1, 3, 8].

Cutaneous testing for mAB hypersensitivity reactions has been shown to be useful for diagnosis and risk stratification in type I and type IV phenotypes [9]. However, test concentrations have not been standardized for most of these drugs. There are few patients tested, skin test reagents are expensive and may be nonaccessible, and nonirritating concentrations need to be confirmed with controls [2].

In our case report, a patient who previously tolerated ustekinumab for nearly 2 years started developing symptoms that did not fit into a specific phenotype. However, the increasing severity of local reactions, paired with immediate and generalized pruritus, raised the suspicion of a potential IgE-mediated reaction. We were able to confirm our suspicions through skin testing and proper validation of nonirritating concentrations in healthy controls. The occurrence of typical type I allergic symptoms during desensitization further corroborated our hypothesis. Despite its perceived safety and only rare reports of allergic reactions to this drug [3], we believe that this is the first published case that validates nonirritating concentrations for skin testing in IgE-mediated reactions to ustekinumab.

Performing skin testing on this patient held significant importance, as it facilitated risk stratification and aided in predicting the likelihood of escalating reactions leading to anaphylaxis. Furthermore, it allowed for better planning of therapeutic schemes, with the cautious use of a desensitization protocol potentially preventing a more severe reaction.

A wider application of these skin test concentrations in other patients treated with ustekinumab (both allergic and nonallergic), paired with complimentary in vitro testing [9], could help with establishing predictive diagnostic values. We hope that skin testing for ustekinumab can prove to be a solid diagnostic and stratification tool in the future.

## Acknowledgements

The authors would like to credit and thank Dr. Ana Paula Pina (Pharmaceutical Department, CHUC, Portugal) for providing ustekinumab and preparing skin test concentrations.

## Conflicts of interest

The authors have no financial conflicts of interest.

## Author contributions

Alves: conceptualization, revision, data collection, data analysis, writing. Cunha: revision, data collection. Mendes and Todo-Bom: conceptualization, revision. Ribeiro: revision, writing, data collection.
